# Linear and circular-polarization conversion in X-band using anisotropic metasurface

**DOI:** 10.1038/s41598-019-40793-2

**Published:** 2019-03-14

**Authors:** M. Ismail Khan, Zobaria Khalid, Farooq A. Tahir

**Affiliations:** 1 Department of Electrical Engineering, COMSATS University Islamabad, Attock Campus, Attock City, Pakistan; 2 Research Institute for Microwave and Millimeter-wave Studies, National University of Sciences and Technology (NUST), Islamabad, Pakistan

## Abstract

An ultrathin single-layer metasurface manifesting both linear cross-polarization conversion (CPC) and linear-to-circular polarization (LP-to-CP) conversion in X-band is presented in this research. The designed metasurface acts as a multifunctional metasurface achieving CPC over a fractional bandwidth of 31.6% (8–11 GHz) with more than 95% efficiency while linear-to-circular polarization conversion is realized over two frequency bands from 7.5–7.7 GHz and 11.5–11.9 GHz. Moreover, the overall optimized structure of the unit cell results in a stable polarization transformation against changes in the incidence angle up to 45° both for transverse-electric (TE) and transverse-magnetic (TM) polarizations. The proposed metasurface with simple structure, compact size, angular stability and multifunctional capability qualifies for many applications in communication and polarization manipulating devices.

## Introduction

Polarization is an essential feature of electromagnetic (EM) waves representing the way in which electric field oscillates at some fixed point in space. Owing to its fundamental role in many polarization sensitive applications and devices such as polarization beam splitters, wave plates, and antennas, researchers have always been curious in designing devices and techniques to control and manipulate polarization of the EM waves. Conventional techniques using Faraday Effect and optical activity of crystals can be used for polarization control, but the limitations associated with these techniques such as bulky size, narrow bandwidth and incidence angle dependent response make them incompatible for many practical applications. The limitations of conventional devices can be overcome through the use of engineered structures called metasurfaces^1^ of whom electric and magnetic responses can be controlled through a judicious design of the subwavelength unit cell.

In this regard, polarization conversion has been realized in microwave^2–4^, terahertz^5,6^, infrared^7^ and visible^8–10^ frequency regimes both in transmission^11–14^ and reflection^15–17^ modes using metasurfaces. Different types of unit cells used in polarization controlling metasurfaces may be broadly categorized as anisotropic^18–22^ and intrinsic^23–25^ or extrinsic^26,27^ chiral unit cells.

Many cross-polarization conversion (*x*-to-*y* or *y*-to-*x* polarization conversion) anisotropic metasurface designs functioning in reflection mode have been reported in literature^28–37^. A broad band (2–3.5 GHz) polarization conversion anisotropic metasurface which is based on multiple plasmonic resonances has been proposed^28^. The bandwidth is further enhanced to 9.1–12.9 GHz by achieving three plasmonic resonances using asymmetric split-ring resonators based metasurface^29^. To improve the bandwidth further, a double head-arrow unit cell based metasurface has been used to exhibit four plasmonic resonances resulting in ultra-wideband (6.2–23.4 GHz) CPC^30^. Similarly, LP-to-CP conversion metasurface based on rectangular loop with a diagonal microstrip are proposed^38^ for operation at 2.5 GHz. Further improvement in the performance is demonstrated by using metallic rectangular-loop arrays based metasurface^39^ that manifests dual-broadband circular-polarization conversion over 5.50–8.94 GHz and 13.1–15.5 GHz. Similarly, wideband (11.4–14.3 GHz) LP-to-CP conversion has been realized using a bilayer metasurface based on orthogonal metallic gratings^40^.

Although the aforementioned designs^28–40^ achieve polarization conversion, however, they work only for one type of operation; either CPC or LP-to-CP conversion. Achieving both linear-cross and LP-to-CP conversion through a single structure has always been desirable. Such a multifunctional device does not only help to miniaturize size of the system by substituting many components through a single multifunctional component but also reduces the overall system complexity and cost. In this regard, in very recent literature^41^, a design has been proposed based on two-corner-cut square multi-ring unit cell achieving both CPC and LP-to-CP conversion in reflection mode, but it works only for normal incidence. Similarly, authors have realized a multifunctional metasurface achieving both CPC and LP-to-CP conversion in transmission mode whereby angular stability is demonstrated only for LP-to-CP conversion^42^. As in practical applications, the impinging wave can strike the metasurface at any oblique angle, therefore, angularly stable response can significantly increase the usefulness of the metasurface in numerous applications.

In this work, we design and practically demonstrate a compact, single layer mirror-symmetric anisotropic metasurface using a novel unit cell having fish-like structure. There are three unusual functionalities exhibited by the proposed metasurface. First, the designed metasurface does not only achieve efficient wideband CPC but also dual-band LP-to-CP conversion in the x-band. Secondly, both type of polarization conversions remain robust to the changes in the oblique incidence angle. Lastly, all these functionalities are accomplished through a simple compact anisotropic design which can be fabricated with less cost.

## Results

### Metasurface Design

In general, any electromagnetic wave reflected from a metasurface consists of two field components, one with same polarization as that of incident wave called co-polarized reflected field and the other is orthogonal component to the incident polarization called cross-polarized reflected field. Let *R*_*lm*_ represents the ratio of the reflected field having polarization *l* to the incident field having polarization *m*. The x and y labels will be used to represent horizontal and vertical linear polarizations while right- and left-handed circular polarizations (RHCP and LHCP) are represented by “+” and “−” respectively. For mathematical analysis of co- and cross- polarizations, the Jones reflection coefficient matrix **R** will be used in Cartesian basis:

1 $${\bf{R}}=(\begin{array}{cc}{R}_{xx} & {R}_{xy}\\ {R}_{yx} & {R}_{yy}\end{array})$$

Reflection coefficients for linear polarization ${R}_{lm}$ (where $l,\,m=x,\,y\,)$ are related to the reflection coefficients for circular polarization ${R}_{lm}$ (where $\,l,\,m=+\,,-$) through the following relation:

2 $$\,{{\bf{R}}}_{{\bf{C}}{\bf{P}}}=(\begin{array}{cc}{R}_{++} & {R}_{+-}\\ {R}_{-+} & {R}_{--}\end{array})=\frac{1}{2}(\begin{array}{cc}{R}_{xx}-{R}_{yy}-i({R}_{xy}+{R}_{yx}) & {R}_{xx}+{R}_{yy}+i({R}_{xy}-{R}_{yx})\\ {R}_{xx}+{R}_{yy}-i({R}_{xy}-{R}_{yx}) & {R}_{xx}-{R}_{yy}+i({R}_{xy}+{R}_{yx})\end{array})$$

A good measure of cross-polarization conversion is polarization conversion ratio (PCR) which is defined for incident *x*-polarization as:

3 $$PCR=\frac{|{R}_{yx}{|}^{2}}{|{R}_{yx}{|}^{2}+|{R}_{xx}{|}^{2}\,}\,$$

For *y*-polarization *x* and *y* are interchanged in Eq. 3. Similarly, the polarization maintaining ability of the metasurface for circular polarizations is determined by polarization maintaining ratio (PMR). For RHCP incident waves, PMR is defined as:

4 $$PMR=\frac{|{R}_{++}{|}^{2}}{|{R}_{-+}{|}^{2}+|{R}_{++}{|}^{2}}\,$$

Similarly, when the incident wave is LHCP then + and − are interchanged in Eq. 4.

The principle of polarization conversion is completely based on anisotropy of the electromagnetic structure. The final optimized unit cell geometry with desired level of anisotropy have been reached step by step through a comprehensive parametric analysis.

**Figure 1:**
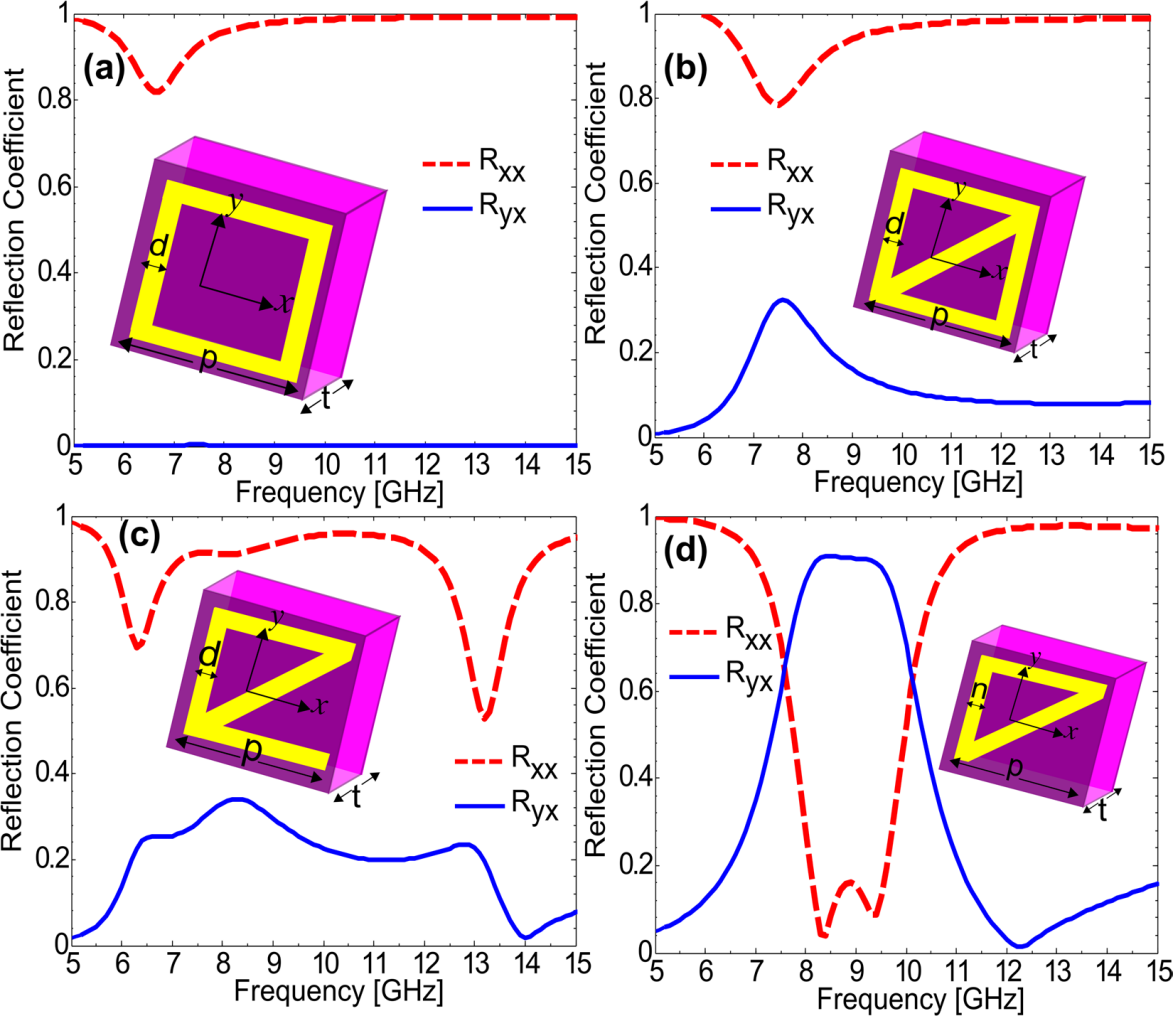
Optimizing unit cell for achieving polarization conversion (**a**) square unit cell (**b**) square unit cell with diagonal metallic strip (**c**) square unit cell with one arm removed while keeping diagonal metallic strip (**d**) half square with diagonal metallic strip or triangular unit cell.

The design evolution of the proposed metasurface is depicted through four major design steps. As a first step, we chose a simple metallic-square to resonate around 7 GHz and analyzed the polarization state of the reflected fields. It can be seen from Fig. 1(a) that this square unit cell does not have any polarization conversion capability as the cross-polarized reflection coefficient ${{\rm{R}}}_{{\rm{yx}}}=0$ while the co-polarized reflection ${{\rm{R}}}_{{\rm{xx}}}$ is maximum. The reason behind this is the isotropic nature of the metallic-square due to which it behaves same to both the orthogonal components of x- or y-polarized incident field. The orthogonal components of the x-polarized field lie at +45° and −45° to the x-axis. It can be seen from Fig. 1(a) that if incident x-polarized field is decomposed into two orthogonal components at ±45° to the x-axis, then it encounters the same structure along both the components. Due to the same structure along both axes, response will also be similar and hence there will be no polarization conversion because polarization conversion requires different phase response along the two orthogonal axes.

This isotropy is broken in Fig. 1(b) due to the diagonal metallic strip inside the square which causes some increase in the cross-polarized reflection coefficient especially at 7.5 GHz where ${{\rm{R}}}_{{\rm{yx}}}=3.5$. Similarly, cross-polarized reflection is further improved, as shown in Fig. 1(c), through increasing anisotropy of the unit cell by removing one side from the square while keeping the diagonal strip as it is. As shown in Fig. 1(d), the cross-polarized reflection is dramatically enhanced in frequency regime 8–9.5 GHz by using half-square with a diagonal strip or triangular unit cell. The reason behind this is different electrical response of the structure along the two orthogonal components (at +45° and −45° to the x-axis) of the x-polarized incident field. The structure behaves as inductor for the component along +45° due to the metallic strip, while as capacitor for the component along −45° due to the gap between the metallic parts of the two unit cells. The design was further optimized for better polarization conversion capabilities by increasing capacitance along axis at −45°.

**Figure 2:**
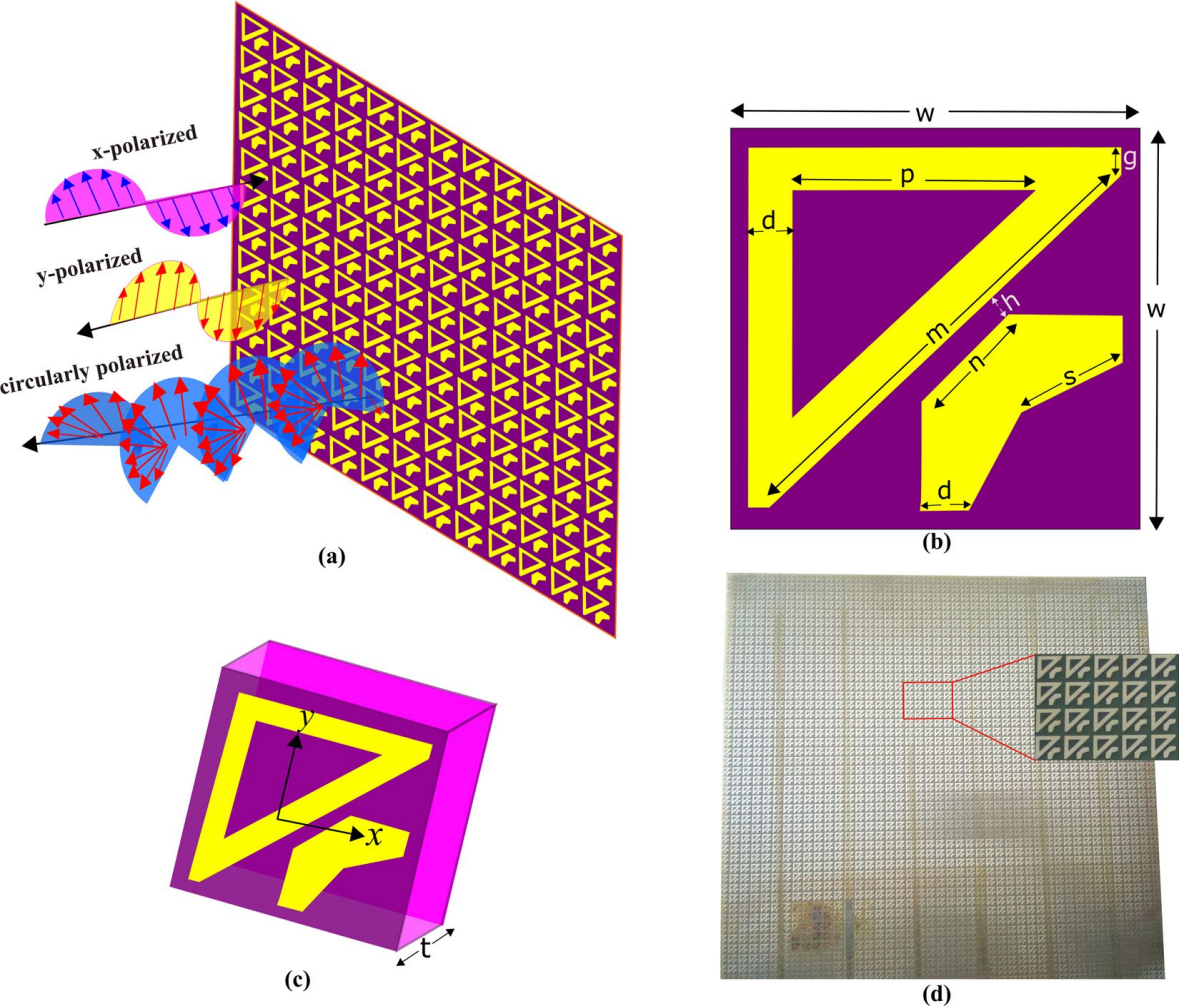
Schematics of the proposed metasurface design (**a**) Two-dimensional array of the unit cells (**b**) Two-dimensional view of the unit cell. Yellow part is the metallic structure (**c**) Three-dimensional view of the unit cell (**d**) Fabricated prototype.

As shown in Fig. 2, the final polarization conversion metasurface consists of two dimensional periodic arrangement of unit cells. The FR4 substrate backed with ground plane was used to model the metasurface. The unit cell, shown in Fig. 2(b), possesses a fish-like structure composed of two disjoint parts. The first part is a right-angled triangular shape while the second part is v-shaped. The optimized dimensions for the unit cell are, in millimeters: w = 7, d = 1, p = 3, s = 1.5, n = 1.5, m = 6.5, g = 0.5, h = 0.5 and thickness of the substrate t = 1.6. The metallic part of the metasurface is made of copper with conductivity 5.8 × 10^7^ S/m while the substrate (FR4) sandwiched between the top and bottom layer has relative permittivity 4.4 and loss tangent 0.02. The unit cell is replicated with periodicity of 7 mm along both *x*- and *y*-axis. Figure 2(d) shows a snapshot of fabricated prototype which has a size of 305 × 305 × 1.6 mm^3^.

**Figure 3:**
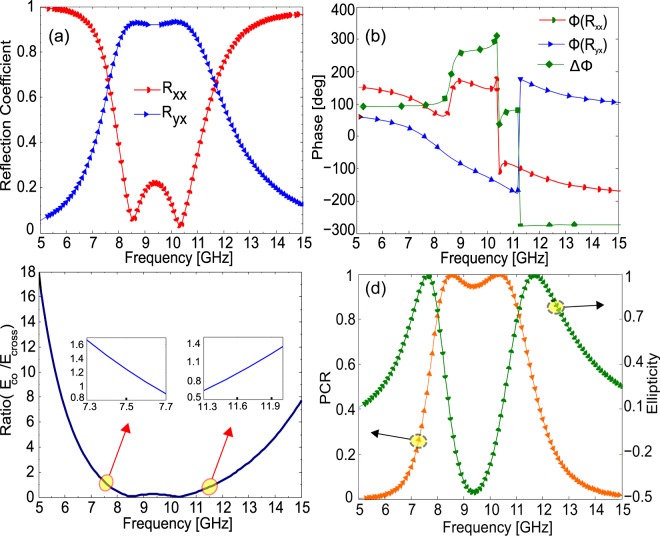
Co- and cross-polarized reflection coefficients for *x*-polarization (**a**) magnitude (**b**) phase (**c**) polarization conversion ratio (**d**) ratio of the co- and cross-polarized reflected fields (on the left axis) and normalized ellipticity (on right axis).

The co- and cross-polarized reflection coefficients of the designed metasurface are shown in Fig. 3(a) for the case when *x*-polarized field,$\,{E}_{i}=\hat{x}{E}_{o}{e}^{ikz}$ is normally incident (at 0°) on the metasurface. It can be seen from Fig. 3(a), that the magnitude of the cross-polarized reflection coefficient $(|{R}_{yx}|)$ is larger than 0.95 while the co-polarized reflection coefficient $(|{R}_{xx}|)$ is negligible in the frequency range 8–11 GHz. Similarly, it can be seen from Fig. 3(d) that PCR is more than 95% in the frequency range 8–11 GHz indicating that the proposed design functions as an efficient polarizing metasurface. Furthermore, the PCR attains its maximum value approaching 1 at resonance frequencies 8.5 and 10.5 GHz.

It can be shown from Eq. 2 that in the frequency band 8–11 GHz, $|{R}_{++}|=|{R}_{--}|=|{R}_{yx}|=|{R}_{xy}|$ while $|{R}_{-+}|=|{R}_{+-}|=0$ which when substituted into Eq. 4 gives $\,PMR=PCR$.

The aforementioned analysis shows that the handedness of the circular polarization is maintained upon reflection from the metasurface in the frequency range 8–11 GHz.

Moreover, Fig. 3(a) shows that the co- and cross-polarized reflection coefficient attain equal magnitude, $|{R}_{xx}|=|{R}_{yx}|\approx 0.7$ at 7.5 and 11.5 GHz while at the same operating frequencies their phase difference, shown in Fig. 3(b), reaches to 90° and −270° respectively. As, magnitude of the co- and cross-polarized reflection coefficient is same while their phase difference is an odd multiple of 90° at 7.5 and 11.5 GHz, therefore, a linearly polarized incident wave is reflected as circularly polarized wave. Similarly, Fig. 3(c) shows that ratio of co- and cross-polarized reflected fields reaches to 1 at 7.5 GHz and 11.5 GHz. The performance of the circular polarizer is acceptable if the ratio of the orthogonal components, co- and cross-polarized fields, lies within 0.85–1.15 and the phase difference is within 85°–95° or −265° to −275°. The frequency range within which this criterion is satisfied will be the operating band of the circular polarizer. As can be seen from Fig. 3(c,d), the given criteria is satisfied over two frequency bands 7.5–7.7 GHz and 11.5–11.9 GHz.

To further investigate whether the reflected wave is right-handed or left-handed circularly polarized, we use Stokes parameters^43^:

5 $$\,{S}_{0}={|{R}_{xx}|}^{2}+{|{R}_{yx}|}^{2},\,{S}_{1}=\,{|{R}_{xx}|}^{2}-{|{R}_{yx}|}^{2},$$

$${S}_{2}=2|{R}_{xx}||{R}_{yx}|cos{\rm{\Delta }}\phi ,{S}_{3}=2|{R}_{xx}||{R}_{yx}|sin{\rm{\Delta }}\phi $$

We define the normalized ellipticity, $e={S}_{3}/{S}_{0}$. Normalized ellipticity is +1 when the reflected wave is right-handed circularly polarized (RHCP) and −1 when it is left-handed circularly polarized (LHCP). It can be seen from Fig. 3(d) that normalized ellipticity is +1 at 7.5 GHz and 11.5 GHz showing that the reflected wave is RHCP.

The response of the proposed design for *y*-polarized incident wave,$\,{E}_{i}=\hat{y}{E}_{o}{e}^{ikz}$ can be found by analyzing mirror symmetry in the unit cell along *v*-axis or $x+y=0$ plane. The mirror operation along this plane can be represented by the matrix:

6 $${\bf{M}}=(\begin{array}{cc}0 & -1\\ -1 & 0\end{array})$$

Owing to the mirror symmetry, the transformation matrix **M** and the reflection coefficient matrix ***R*** satisfy ${\boldsymbol{MR}}{{\boldsymbol{M}}}^{-1}=R\,$, which after some simple algebraic steps, gives ${R}_{xx}={R}_{yy}\,\,$and $\,{R}_{yx}={R}_{xy}\,$. Also, since the unit cell has same physical structure along *x*- and *y-*axis, therefore, the metasurface will give similar response both to *x*- and *y*-polarizations.

**Figure 4:**
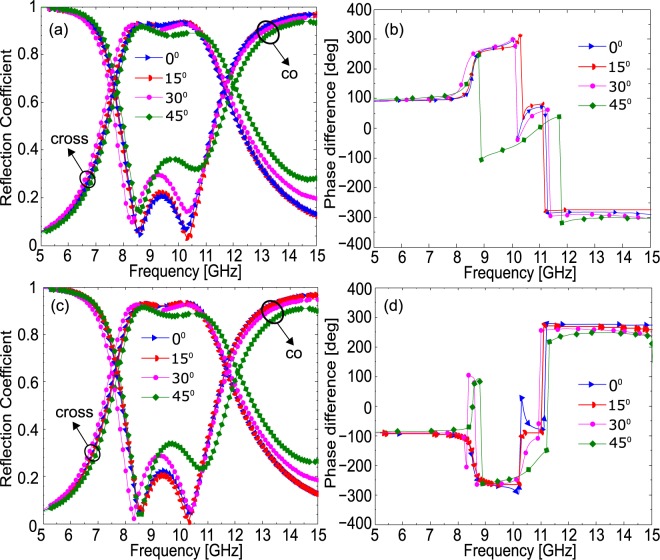
(**a**) Magnitude of co- and cross-polarized reflection coefficients and (**b**) Phase difference for TM polarization). (**c**) Magnitude of co- and cross-polarized reflection coefficients and (**b**) Phase difference for TE polarization.

In real world applications, the incident EM waves can strike the structure at any oblique angle, therefore, a stable response against the deviations in the incidence angle will significantly increase the applicability of the metasurface. To investigate the angular stability of the proposed design, simulations were carried out both for transverse-magnetic (TM) and transverse-electric (TE) polarizations under oblique incidence as shown in Fig. 4(a,b) and Fig. 4(c,d) respectively. As can be seen from Fig. 4(a), the proposed design gives stable polarization transformation against changes in the incidence angle up to 45°, not only in cross-polarization conversion frequency regime (8–11 GHz) but also at 7.5–7.7 GHz and 11.5–11.9 GHz where circular-polarization conversion is achieved. The proposed design manifests stable polarization transformation capabilities not only for *x*-polarization (TM) but also for *y*-polarization (TE) as shown in Fig. 4(c,d). The stable polarization conversion of the metasurface is realized through the compact subwavelength unit cell size (0.26λ_o_), small substrate thickness (0.06 λ_o_) where λ_o_ is the free space wavelength at 11.5 GHz and optimized structure of the unit cell.

## Theoretical Analysis

To understand cross-polarization conversion, we need to find eigen-polarizations and eigenvalues for the proposed design. To carry out this analysis, we ignore the dielectric losses in the subsequent discussion for simplicity. From the results shown in Fig. 3, it can be seen that at resonance frequency 8 GHz, $|{R}_{xy}|=|{R}_{yx}|\approx 1\,$and $|{R}_{xx}|=|{R}_{yy}|\approx 0$ which when substituted in reflection coefficient matrix gives:

7 $${\bf{R}}=(\begin{array}{cc}0 & 1\\ 1 & 0\end{array})\,$$

**Figure 5:**
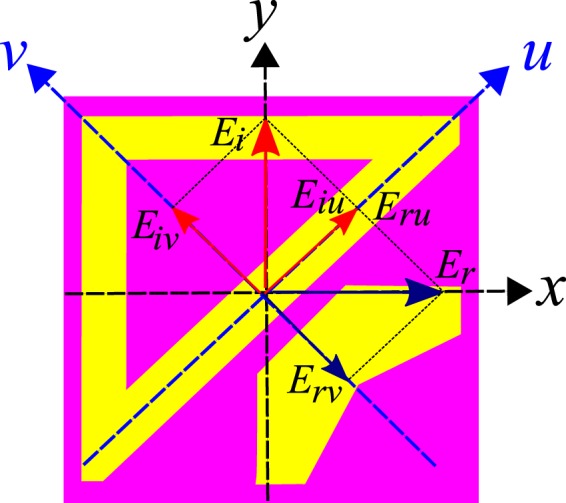
Polarization conversion of *y*-polarized field into *x*-polarized field, *y*-polarized electric field is resolved into two orthogonal components along *u*- and *v*-axis, *u*-component is reflected in phase while *v*-component is reflected out of phase resulting into reflected field along *x*-axis.

As can be easily checked, the linearly independent eigenvectors for matrix **R** are $${\boldsymbol{u}}={(\begin{array}{cc}1 & 1\end{array})}^{T}$$ and $${\boldsymbol{v}}={(\begin{array}{cc}-1 & 1\end{array})}^{T}$$ with eigenvalues ${e}^{i0}=1\,\,$and ${e}^{i\pi }=-\,1$ respectively. Physically this means that *u*- and *v*-polarized incident waves are reflected with unity magnitude and ${0}^{0}$ and ${180}^{0}$ phase respectively without any cross-polarization conversion. Since, no cross-polarization conversion takes place for *u-* and *v*-polarizations, therefore, $|{R}_{uu}|=|{R}_{vv}|\approx 1\,\,$and $|{R}_{uv}|=|{R}_{vu}|\approx 0.$ Now, consider a normally incident *y*-polarized electromagnetic wave ${{\boldsymbol{E}}}_{{\boldsymbol{i}}}=\hat{y}{E}_{i}{e}^{ikz}$ with wave number *k* striking the metasurface, as shown in Fig. 5.

There are two coordinate systems used in Fig. 5: *xy* and *uv* coordinate system where *u* and *v*-axis are oriented at +45° to the *x*- and *y*-axis respectively. As can be seen from Fig. 5, the unit cell possesses anisotropy along *u*- and *v*-axis. Moreover, the structure has mirror symmetry along *v*-axis. The incident electric field, ${{\boldsymbol{E}}}_{{\boldsymbol{i}}}=\hat{y}{E}_{i}{e}^{ikz}$, can be resolved into two orthogonal *u* and *v* components, ${{\boldsymbol{E}}}_{{\boldsymbol{i}}}=\hat{y}{E}_{i}=\hat{u}{E}_{iu}+\hat{v}{E}_{iv}$, at $z=0$ where ${E}_{iu}={E}_{iv}=0.707{E}_{i}$. As *u*- and *v*-polarized components, ${E}_{iu}$ and ${E}_{iv},$ are reflected with same magnitude, ${E}_{ru}={E}_{rv}={E}_{r}\,,$ and 0° and 180° phase respectively, therefore, the reflected field becomes:

8 $$\,{{\boldsymbol{E}}}_{{\boldsymbol{r}}}=\hat{u}{E}_{r}-\hat{v}{E}_{r}=\hat{x}{E}_{r}$$

So, the reflected field is along *x*-axis and hence cross-polarization conversion occurs. This can be also understood pictorially from Fig. 5, where ***E***_*r*_, obtained from the vector addition of ${E}_{ru}$ and $\,{E}_{rv}\,$, is along *x*-axis.

Although, in above discussion, phase of the reflected *u*-component ${\varphi }_{uu}$ was 0° while for *v*-component $\,{\varphi }_{vv}={180}^{{\rm{o}}}$, but, in general scenario, the phase difference should be 180°, i.e. ${\rm{\Delta }}\varphi ={\varphi }_{uu}-{\varphi }_{vv}={180}^{{\rm{o}}}$, regardless of the absolute values of ${\varphi }_{uu}$ and $\,{\varphi }_{vv}$. So, the general requirements for CPC is that the orthogonal components of the incident field must be reflected with unity magnitude and 180° relative phase difference. To prove this, consider a *y*-polarized incident electric field, ${{\boldsymbol{E}}}_{{\boldsymbol{i}}}=\hat{y}{E}_{i}=\hat{u}{E}_{iu}+\hat{v}{E}_{iv}$ at $z=0\,\,$where $\,{E}_{iu}={E}_{iv}=0.707{E}_{i}$. The reflected electric field becomes:

9 $$\,{{\boldsymbol{E}}}_{{\boldsymbol{r}}}=\hat{u}({R}_{uu}{E}_{iu}{e}^{i{\phi }_{uu}}+{R}_{uv}{E}_{iv}{e}^{i{\phi }_{uv}})+\hat{v}({R}_{vv}{E}_{iv}{e}^{i{\phi }_{vv}}+{R}_{vu}{E}_{iu}{e}^{i{\phi }_{vu}})$$

As, $|{R}_{uu}|=|{R}_{vv}|\approx 1\,\,$and $|{R}_{uv}|=|{R}_{vu}|\approx 0$ and, therefore Eq. 9 gives:

10 $${{\boldsymbol{E}}}_{{\boldsymbol{r}}}=\hat{u}{E}_{iu}{e}^{i{\phi }_{uu}}++\,\hat{v}{E}_{iu}{e}^{i{\phi }_{vv}}$$

Now, in order to be orthogonal to the incident electric field, the reflected field must satisfy $\,{{\boldsymbol{E}}}_{{\boldsymbol{i}}}.{{\boldsymbol{E}}}_{{\boldsymbol{r}}}=0$, which gives:

11 $$\,{E}_{iu}{e}^{i{\phi }_{uu}}(1+{e}^{i({\phi }_{vv}-{\phi }_{uu})})=0\,$$

Eq. 11 has nontrivial solution if and only if ${\phi }_{vv}-{\phi }_{uu}=\pi$ which completes the proof.

To find eigenvalues and eigenvectors for LP–to-CP conversion, consider the reflection coefficient matrix at 7.5 GHz obtained from the results shown in Fig. 3.

12 $${\bf{R}}=0.707(\begin{array}{cc}{e}^{i\frac{\pi }{2}} & 1\\ 1 & {e}^{i\frac{\pi }{2}}\end{array})$$

The linearly independent eigenvectors for matrix **R** are $${\boldsymbol{u}}={(\begin{array}{cc}1 & 1\end{array})}^{T}$$ and $${\boldsymbol{v}}={(\begin{array}{cc}-1 & 1\end{array})}^{T}$$ with eigenvalues ${e}^{i\frac{\pi }{4}}\,\,$and ${e}^{i\frac{3\pi }{4}}$ respectively. Physically, this means that if incident electric field is polarized along *u*- or *v*-axis then this is reflected with unity magnitude and a phase delay of 45° and 135° respectively leading to a phase difference of 90°. Since, *u*- and *v*-polarization are the eigen-polarizations, therefore, they are reflected from the metasurface without any polarization transformation.

The previous discussion shows that the phase difference of the orthogonal reflected *u*- and *v*-components determine whether CPC or LP-to-CP conversion is achieved. The root cause of the different phase along *u*- and *v*-axis is the anisotropy of the unit cell. Along *u*-axis, the unit cell acts as an inductor with inductance L because of the metallic strip. On the other hand, there are gaps between metallic strips along *v*-axis which gives capacitive effects and hence the unit cell behave as a capacitor with capacitance C. The inductive and capacitive effects along *u*- and *v*-axis respectively cause different phases for the corresponding polarizations and hence lead to polarization conversion.

**Figure 6:**
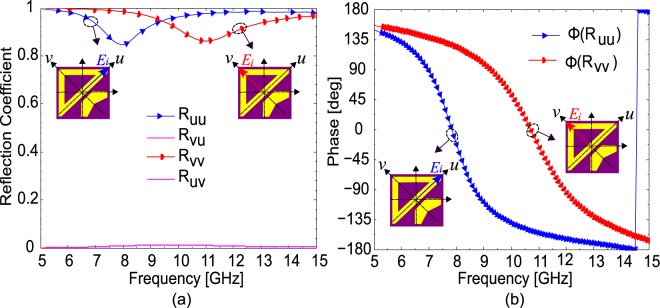
(**a**) Magnitude of the reflection coefficients and (**b**) phase for *u*- and *v*-polarization.

To verify the above theoretical analysis, the proposed design is simulated for *u-* and *v-*polarized incident waves. As shown in Fig. 6, the magnitude of the cross-polarized reflection coefficients, $|{R}_{uv}|\,\,$and $\,|{R}_{vu}|$, is almost zero while the co-polarized reflection coefficients, $|{R}_{uu}|\,\,$and $\,|{R}_{vv}|$, have magnitudes larger than 0.9 for most of the frequencies. Similarly, Fig. 6(b) shows that the phase difference between *u*- and *v*-polarized reflected fields at 7.5 and 11.5 GHz is 90° while it is almost 180° in frequency range 8–11 GHz. As, *x*- or *y*-polarized fields can be resolved into *u*- and *v*-components, ${E}_{x}\hat{x}=0.707({E}_{x}\hat{u}-{E}_{x}\hat{v})$ and $\,{E}_{y}\hat{y}=0.707({E}_{y}\hat{u}+{E}_{y}\hat{v})$, therefore, after reflection from the metasurface, *u*- and *v*-components have same magnitude and 180° or 90° phase difference resulting into CPC or LP-to-CP conversion respectively.

### Frequency Tunability

**Figure 7:**
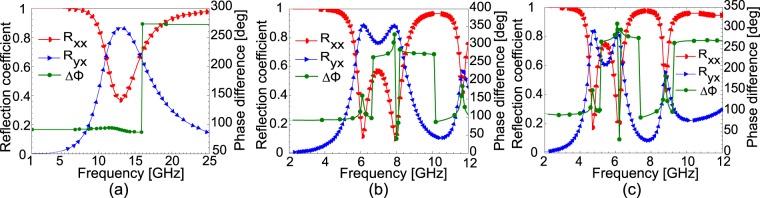
(**a**–**c**) Magnitude and phase difference of the reflection coefficients when physical dimensions in *xy*-plane are scaled by (**a**) 0.5 (**b**) 1.5 and (**c**) 2.

In order to find applications in other frequency bands, one must show how to shift the operating band of the proposed design to lower or higher frequencies by adjusting the physical dimensions of the unit cell. To examine this, the proposed design is simulated with different physical parameters and the results are shown in Fig. 7. It is clear from Fig. 7(a) that the working frequency of the metasurface for LP-to-CP conversion is increased from 7.5 and 11.5 GHz to 11 and 16 GHz respectively when the physical parameters of the unit cell in the *xy*-plane are decreased through scaling by 0.5. Moreover, the working frequency band for CPC also shifts to 11.5–15.5 GHz from 8–11 GHz. Similarly, Fig. 7(b) shows that when the dimensions of the unit cell in the *xy*-plane are scaled by 1.5, the operating frequencies for LP-to-CP conversion decreases to 5.5 and 8.5 GHz while the CPC band shifts to 6–8 GHz. In the same fashion, Fig. 7(c) shows that operating frequencies, 7.5 and 11.5 GHz, for LP-to-CP conversoin are further decreased to 4.5 and 6.5 GHz respectively when the dimensions of the unit cell are increased through scaling by 2. It can be deduced from the above parameteric analysis that the operating bands can be shifted to higher or lower frequencies by scaling the unit cell by a number less or greater than 1 respectively in the *xy*-plane. Thus, the same proposed design may be further optimized to achieve CPC and LP-to-CP conversions in terahertz, infra-red and visible frequency regimes.

**Figure 8:**
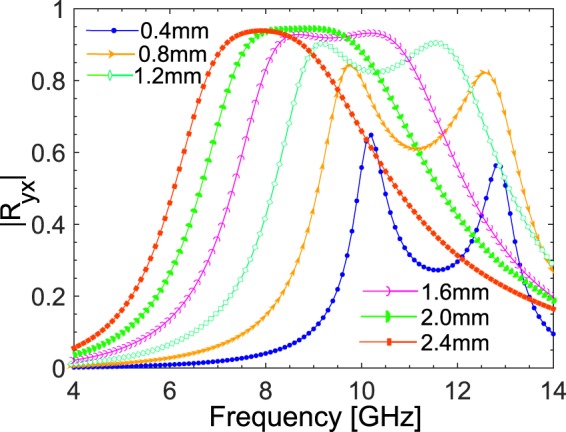
Variation of the cross-polarized reflection coefficient with substrate thickness.

To see the effect of substrate thickness on the response of the metasurface, simulations were carried out for the proposed design under different substrate thickness values from 0.8 mm to 2.4 mm. As is clear from Fig. 8, optimum response is obtained for thickness of 1.6 mm. Moreover, the response of the metasurface shifts towards lower frequencies as the substrate thickness is increased. This occurs due to the scale invariance of Maxwell equations where wavelength to thickness ratio (*λ*/*t*) is maintained by shifting the response to longer wavelengths (smaller frequencies) for large substrate thicknesses.

## Experiment

**Figure 9:**
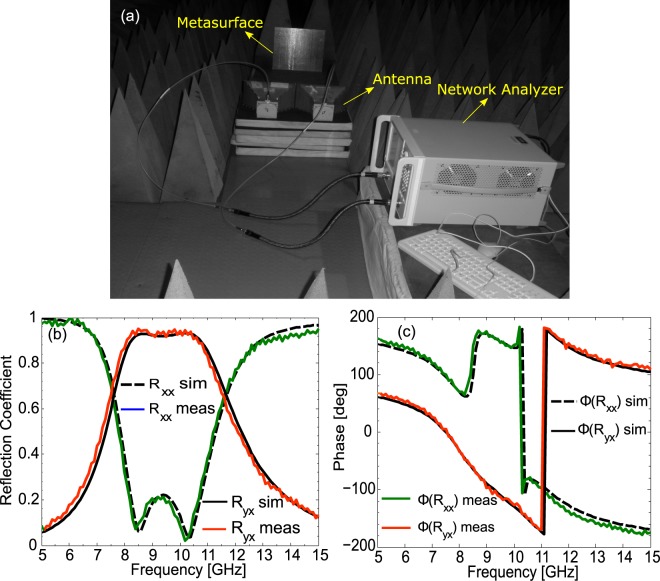
(**a**) Measurement setup (**b**) Magnitude of the simulated and measured co- and cross-polarized reflection coefficients. (**c**) Simulation and measurement results for the phase.

In order to verify the simulation results through experimental measurements; the designed metasurface is fabricated on a 305 × 305 × 1.6 mm^3^ FR4 sheet backed by copper cladding. The fabricated prototype consists of 44 × 44 unit cells etched out using standard PCB techniques. Wideband horn antennas manufactured by EMCO with a working frequency band of 1–18 GHz are utilized for irradiating the surface and then receiving the reflected waves. The received signal strength is measured by Agilent vector network analyzer N5232A. A snapshot of the constructed measurement platform is shown in Fig. 9(a). For the measurements of the co-polarized reflection coefficients, both antennas are positioned along the same orientation, either horizontal (for *x*-polarization) or vertical (for *y*-polarization). On the other hand, for measuring cross-polarized reflections one antenna is placed horizontal and the other is placed vertical. Figure 9(a,b) show simulated and measured results (after necessary calibration) for the magnitude and phase of reflection coefficients when the impinging wave is *x*-polarized. As shown in Fig. 9, simulation and measurement results are consistent. The small discrepancies arising between measurements and simulations are caused by imperfections in the fabrication process and small size of the prototype.

## Discussion

We designed and experimentally validated a compact single layer reflective metasurface achieving single wideband cross-polarization conversion and dual-band linear-to-circular polarization conversion. The anisotropy of the unit cell makes the metasurface achieve cross-polarization conversion over frequency band 8–11 GHz (31.6% fractional bandwidth) by retarding one component of the reflected wave through a phase of 180° with respect to the other orthogonal component. Similarly, linear-to-circular polarization conversion and vice versa is demonstrated over two frequency bands 7.5–7.7 and 11.5–11.9 GHz by introducing a phase delay of 90° between the orthogonal components. Eigen-polarization analysis was carried which showed that no cross-polarization conversion takes place when the electric field of the incident wave is oriented at ±45 degree to the *x*- or *y*-axis. Furthermore, the overall optimized structure of the unit cell results in a stable response for oblique incidence angle up to 45° both for transverse-electric and transverse-magnetic polarizations. The proposed design is fabricated and the simulation results are verified through experimental measurements.

## Methods

The HFSS software is used for full-wave numerical simulations of proposed metasurface. In the simulation setup, the unit cell is placed in *xy*-plane where unit-cell boundary conditions are imposed. EM waves of different polarizations are incident on the unit cell from the top port and the corresponding S-parameters for magnitude and phase are obtained. Moreover, the dielectric used in the unit cell is FR4 with relative permittivity 4.4 and loss tangent 0.02 while the material used for metallic part is copper with conductivity 5.8 × 10^7^ S/m. The standard printed circuit board (PCB) techniques were applied to fabricate the proposed metasurface by imprinting 44 × 44 unit cells on the top of 305 × 305 × 1.6 mm^3^ FR4 substrate. Two double-ridged wideband horn antennas manufactured by EMCO with a working frequency band of 1–18 GHz were used for transmitting and receiving EM waves. In order to measure S-parameters, Agilent vector network analyzer N5232A was utilized. Metasurface was placed in the far field at the same height level as that of transmitting and receiving antennas. All measurements were carried out inside anechoic chamber. In order to measure co-polarized reflection coefficients, both transmitting and receiving antennas are positioned along the same orientation either horizontal or vertical while for cross-polarized coefficients they are oriented perpendicular to each other. It is important to note that some energy will be diffracted due to the finite size of the fabricated sample and hence will not be received by the receiving antenna. In order to take this into consideration, we used a simple copper plate of the same size as that of metasurface to compare the results.

## Data Availability

The datasets generated during and/or analyzed during the current study are available from the corresponding author on reasonable request.
